# Extraction and Characterization of Organ Components of the Malaysian Sea Cucumber* Holothuria leucospilota* Yielded Bioactives Exhibiting Diverse Properties

**DOI:** 10.1155/2019/2640684

**Published:** 2019-04-15

**Authors:** Abdoulie Ceesay, Mariana Nor Shamsudin, Mohammed Aliyu-Paiko, Intan Safinar Ismail, Muhammad Farhan Nazarudin, Norfarrah Mohamed Alipiah

**Affiliations:** ^1^Laboratory of Marine Biotechnology, Institute of Bioscience, Universiti Putra Malaysia, 43400 UPM Serdang, Selangor, Malaysia; ^2^Biochemistry Department, Faculty of Natural Sciences, Ibrahim Badamasi Babangida (IBB) University, P. M. B. 11 Lapai, Nigeria; ^3^Chemistry Department, Faculty of Science, Universiti Putra Malaysia, 43400 UPM Serdang, Selangor, Malaysia

## Abstract

The aim of the present study was to extract and characterize bioactive components from separate body organs of* Holothuria leucospilota*. Preliminary qualitative assessment of the crude extracts was positive for phenols, terpenoids, carbohydrates, flavonoids, saponins, glycosides, cardiac glycosides, steroids, phlobatannins, and tannins in all body organs evaluated. Phenolics were the most abundant group of bioactives accounting for approximately 80%. The extraction solvent mixtures that yielded most compounds evaluated were methanol/acetone (3:1, v:v) and methanol/distilled water (3:1, v:v). In other analyses, GC-MS data revealed diverse metabolic and biologically active compounds, where those in high concentrations included 2-Pentanone, 4-hydroxy-4-methyl- among the ketones; phenol- 2,4-bis(1,1-dimethylethyl)-, a phenol group; and 2-Chlorooctane, a hydrocarbon. Among FA and their methyl/ethyl esters, n-hexadecanoic acid, 5,8,11,14-eicosatetraenoic acid ethyl ester (arachidonic acid), and 5,8,11,14,17-eicosapentaenoic acid methyl ester (EPA) were among the most abundant FAMEs accounting for approximately 50% of the subgroups measured. Data from GC-FID analysis revealed methyl laurate (C12:0), methyl myristate (C14:0), methyl palmitate (C16:0), and methyl stearate (18:0) methyl esters as the most abundant saturated FA, whereas* cis*-9-oleic methyl ester (C18:1) and methyl linoleate (C18:2) were found as the major monounsaturated FA and PUFA FAMEs, respectively, in the body wall of the species. Taken together, the extraction and characterization of different categories of metabolically and biologically active compounds in various organ extracts of* H. leucospilota *suggest that the species is potentially a rich source of cholesterol-lowering, antioxidant, antimicrobial, and anticancer agents. These substances are known to benefit human health and assist in disease prevention. These findings justify the use of sea cucumbers in traditional folklore medication and the current interest and attention focused on the species to mine for bioactives in new drugs research.

## 1. Introduction

Sea cucumbers are high value marine organisms that are found well distributed worldwide. Expert estimate suggests that there are presently about 1, 400 species of sea cucumbers and new species continue to be identified and classified among six valid orders: Apodida, Aspidochirotida, Elasipodida, Molpadiida, Dendrochirotida, and Dactylochirotida [1]. For centuries, different species of sea cucumbers have been fished primarily for food [2], whereas products of biomedical importance from them have also been used locally and internationally in traditional medications [3].

As food, many species of edible sea cucumbers have high nutritional value because of their content of high-quality proteins, which comprise a rich profile of amino acids and low level of fat that is supported by a rich combination of desirable trace minerals. In summary, the species is highly regarded in Asia as a rare traditional delicacy, an important traditional medication, and aphrodisiac dating back many centuries [4]. To highlight the historical importance of these organisms in the Far East, [5] opined that sea cucumbers have been useful in folk medicine, where data in the literature suggests that about fifty-two species are commercially exploited as food, and most of these are native to the tropics and subtropics [6].

In addition to consumption as functional food, results of published studies also suggest that various organs of sea cucumbers contain numerous bioactives that show biological activity, which may have potential as therapeutic agents in human medicine and for veterinary applications. Some important compounds already identified in these marine species include sulfated polysaccharides such as fucosylated chondroitin sulfate [7], sterols, sterol glycosides, frondogenin and its glycosides [8, 9]. Furthermore, lectins, heparin, cerebrosides, gangliosides, omega-6, and omega-3 fatty acids (n-6 and n-3 FA) that are known to exhibit multiple biological activities in living cells are also among the substances isolated from sea cucumbers. These compounds are now in use in anticancer, antiangiogenic, anticoagulant, antihypertensive, anti-inflammatory, antimicrobial, antifungal, antioxidant, antithrombotic, and antitumor applications [8].

Among the species of sea cucumber so far evaluated,* Holothuria leucospilota *is particularly widely distributed in the shallow reefs of tropical, subtropical, and Indo-Pacific regions, including the Red Sea [10–12]. They are also found living in the Pacific Ocean, from Northern Australia southwards to Lord Howe, and have been discovered in the tropical waters of the Indo-West Pacific feeding on phytoplankton, micro- and macroalgae, and bacteria [13]. Analysis of* H. leucospilota *has revealed some unique additional bioactive compounds that are of therapeutic significance, including phenolic compounds [14], essential and free fatty acids [15, 16], gangliosides [17], and carotenoids [18] among many others. In other words, the detection of these compounds in the species has been considered as strong evidence to support the use and application of extracts from* H. Leucospilota *in folklore medicine, to treat several infections and diseases.

Despite this relative advantage, majority of studies encountered on the species in the literature are primarily focused on its content of triterpene glycosides or some other specific compounds of interest to researchers. The complete analysis of bioactives in the species is yet to be carried out, suggesting that more quantitative research is required, to evaluate the entire body organs of* H. leucospilota *for bioactive components. The present study was therefore aimed at fully extracting and characterizing the complete bioactive components in the body organs of adult* H. leucospilota *sampled from the coastal shores of Malaysia, in order to determine the metabolically and biologically active substances and the fatty acid (FA) constituents.

## 2. Materials and Methods

### 2.1. Source and Preparation of Sea Cucumber Samples

Sea cucumbers used in the present study were sampled from Teluk Kemang, near Port Dickson (2° 27′ 46.61′′N 101° 50′ 42.98′′E), Malaysia. Live, adult samples were collected by hand from the sea shores in plastic sample containers during low tides. Samples were washed free of debris with clean sea water, kept in transparent polyethylene bags in chilled sampling boxes, and transported to the laboratory facilities of Marine Biotechnology, Institute of Biosciences (IBS), Universiti Putra Malaysia (UPM), Serdang, Selangor, Malaysia.

In the laboratory, live* H. leucospilota* samples were further cleaned of sand, salt water, and left-over debris with distilled water and then cut into small pieces (2 cm × 2 cm) with sterile knives, forceps, and other dissecting tools. The body wall, Cuvierian tubules, and visceral organs were separated, washed, and kept in separate sample bottles. Separated organ samples were then lyophilized using a bench top, K-manifold freeze-dryer (FreeZone, Labconco, USA) for 120 hours. The freeze-dried samples were then ground with a blender (Magic Bullet, Model MBR-1101G, USA) to fine powder, quantified on electronic weighing balance (Model B12-FA/JA, Taiwan), and kept in dry sample bottles. The dry powdered samples in bottles were covered with aluminum foil papers and stored at -20°C until used during subsequent analyses, within three weeks. The powdered samples were appropriately labeled and identified as body wall (BW), Cuvierian tubules (CT), and visceral organs (VO) in subsequent evaluations.

### 2.2. Crude Sample Extraction Procedure

Extraction of bioactive constituents was performed on dry powdered* H. leucospilota* organ samples, using Soxhlet and conventional solvent extraction techniques. Briefly during the Soxhlet extraction, known volumes (v:v, label) of analytical grade solvents, methanol/acetone (3:1, MA), isopropanol/acetone (3:1, PA), ethanol/distilled water (3:1, ED), methanol/distilled water (3:1, MD), and iso-propanol/distilled water (3:1, PD), were used. Repeated extraction was carried out on each separately weighed sample per solvent mixture, to extract bioactive constituents from various powdered sea cucumber organ samples. Each sample extraction procedure lasted about 5 hours. Subsequently, fractions from each repeated solvent extraction were pooled together and filtered with Whatman (No. 1) filter paper, and the filtrate was evaporated to dryness on a rotary evaporator.

In the conventional solvent extraction procedure, the powdered organ samples were weighed and dissolved in analytical grade (v:v, label) acetone (A) and methanol/acetone (1:1, MA). Approximately 200 g of BW, 70 g CT, and 73 g VO were extracted in 2 L, 700 mL, and 730 mL of extraction solvent, respectively (representing a concentration of 1 g of organ sample in 10 mL of extraction solvent). Extraction was carried out under ambient temperature for 4 days with constant shaking, according to slight modification to the method described by [20]. All extractions were carried out under aseptic conditions, in clean dry beakers. The procedure was repeated many times (between twelve to fifteen cycles) until no further color changes were observed in the solvents. Batches of the extracts for each sample were then pooled and filtered, and the filtrate was concentrated on a rotary evaporator, as previously described. Water (moisture) was removed from the extracts by freeze-drying and the dry organ extracts (BW, CT, and VO) were then used in subsequent evaluations immediately or stored at -20°C until used.

### 2.3. Qualitative Characterization of Bioactives in H. leucospilota Crude Organ Extracts

The screening procedure used to characterize the bioactives in crude organ extracts of* H. leucospilota *was carried out according to slight modification to that adopted by [21]. In this analytical method, the presence of bioactive compounds that contribute to flavor, color, and other important characteristics in the organs of sea cucumber was determined.

To identify groups of compounds in the crude organ extracts, however, standard chemical screening was performed according to the procedure published by other authors [22–25]. Briefly, flavonoids were screened using FeCl_3_ via Pew's and Shinoda's tests, glycosides were determined by Keller- Killani test, saponins were evaluated by frothing test, tannins were evaluated by FeCl_3_ and lead acetate, terpenoids were evaluated by Salkowski's test, carbohydrates were determined following Barfoed's, Fehling's, and Molisch's tests, and steroids were evaluated by Liebermann-Burchard test. The tests for phenolics, phlobatannins, and cardiac glycosides were carried out in accordance with the procedure reported in the literature [22–25].

### 2.4. Quantitative Characterization for Bioactive Groups in H. leucospilota Organ Extracts

#### 2.4.1. Determination of Total Phenolics Content

Total phenolics content (TPC) was determined using the Folin-Ciocalteu reagent, according to slight modification of the method published by [26]. To determine TPC, 0.5 mL of dry BW crude extract (50 mg/mL) was added to 2.5 mL of Folin-Ciocalteu reagent diluted with distilled water (1:10, v:v) and 1.25 mL of 20% aqueous sodium carbonate in a test tube covered with aluminum foil. The test tube was vortex-mixed and incubated at ambient temperature (28°C) for 40 minutes. The absorbance of the mixture was then measured at 725 nm in a spectrophotometer (Shimadzu UV-1601 UV-VIS visible, Japan), against a blank containing the same mixture, except that Folin-Ciocalteu reagent was replaced with distilled water. TPC for the extract was calculated from an equation obtained from a calibration curve generated with solution of gallic acid dissolved in methanol and expressed as gallic acid equivalents (GAE) per gram of extract. The content of phenolics in gallic acid equivalent was calculated using the following formula:

(1) $$\begin{array}{c} C=\frac{\left(C\times V\right)}{m} \end{array}$$

where C is the total content of phenolic compounds, mg/g dry extract in GAE; c is the concentration of gallic acid equivalent from the calibration curve, *μ*g/mL; V is the volume of extract used, mL; and m is the dry weight of extract, g.

#### 2.4.2. Determination of Total Content of Flavonoids

Total flavonoids content (TFC) was determined using aluminum chloride colorimetric assay, following slight modifications to the procedure reported by [27]. TFC was carried out by adding 0.5 mL of the BW crude extract (50 mg/mL) to 0.3 mL of NaNO_2_ (5%) in a test tube covered with aluminum foil and mixing. After incubation for 5 minutes, 0.3 mL of AlCl_3_ (10%) was added into the test tube and further incubated for 1 minute. Approximately 2 mL of NaOH (1M) and 1.4 mL of distilled water were added to the mixture and vortex-mixed before the absorbance was measured at 510 nm in a spectrophotometer. TFC for each extract was calculated from the equation obtained from a calibration curve generated with a solution of rutin dissolved in methanol, where TFC was expressed as rutin equivalents (RE) per gram of extract. The content of flavonoids in rutin equivalent was calculated by the following formula:

(2) $$\begin{array}{c} C=\frac{\left(C\times V\right)}{m} \end{array}$$

where C is the total content of flavonoid compounds, mg/g dry extract, in RE; c is the concentration of rutin equivalent established from the calibration curve, *μ*g/mL; V is the volume of extract used, mL; and m is the dry weight of extract, g.

#### 2.4.3. Determination of Total Content of Saponins

Total saponins content (TSC) was determined according to minor modifications to the vanillin-sulphuric acid calorimetric method, as reported by [28]. To determine TSC, 0.25 mL of the BW crude extract (50 mg/mL) was added to 0.25 mL of vanillin reagent (8%) and 0.25 mL of 72% H_2_SO_4_ in a test tube covered with aluminum foil. The test tube was vortex-mixed and warmed on a hot water bath maintained at 60°C. After 10 minutes, the sample mixture was cooled in ice cold water for 4 minutes and the absorbance was read at 544 nm, using a spectrophotometer. TSC for each extract was calculated from the equation obtained from a calibration curve generated with a solution of diosgenin dissolved in methanol and the TSC was expressed as diosgenin equivalents (DE) per gram of extract. The content of saponins in diosgenin equivalent was calculated by the following formula:

(3) $$\begin{array}{c} C=\frac{\left(C\times V\right)}{m} \end{array}$$

where C is the total content of saponin compounds, mg/g dry extract, in DE; c is the concentration of diosgenin established from the calibration curve, *μ*g/mL; V is the volume of extract used, mL; and m is the dry weight of extract, g.

### 2.5. GC-MS Analysis to Characterize and Quantify Bioactives in H. leucospilota Extracts

Freeze-dried sea cucumber organ extracts were characterized quantitatively via Gas Chromatography-Mass Spectrometry (GC-MS), according to slight modifications to the method adopted by [29], using a Shimadzu QP5050A GC-MS system. In the experimental procedure, 1.0 *μ*L of sample was separated on a Zebron (ZB-FFAP GC-17A, version 3, USA) column (30 m × 0.25 mm). Splitless injection was performed using a purge time of 1 minute. Helium represented the carrier gas at a flow rate of 1mL/min. The column temperature was maintained at 50°C for 3 minutes, then programmed at 250°C for 10 minutes, and maintained at 250°C for 30 minutes. The inlet and the detector temperatures were set at 250°C and the solvent cut time was set at 4.50 minutes. Identification of peaks was based on a computer-based program matching the mass spectra with those in the library for National Institute of Standards and Technology (NlST 08 and NIST 08s). This was done by comparing retention time data with that obtained for authentic laboratory standards. Individual detected peak areas were quantified and expressed as percentage of total components detected.

### 2.6. Quantitative Fatty Acids Analysis via GC-FID

Extracted lipids from dry BW samples were esterified to form fatty acid methyl esters (FAMEs) according to slight modifications to the method used by [30]. Approximately 0.2 g of extracted lipid samples was diluted with 4 mL of hexane, followed by addition of 0.2 mL of sodium methoxide in sealed tubes. The mixture was vortex-mixed and left to stand for about 30 minutes to form two layers, subsequent to which the top layer containing FAMEs was collected for fatty acids (FA) analysis. Gas chromatography (GC) (model COND BPX70.M) fitted with a capillary column (BPX7060 m × 0.25 mm) and equipped with flame ionization detector (FID) was used. Approximately 1 *μ*L of each* H. leucospilota *organ extract was injected and separated on the GC column. Splitless injection was also performed at 10-minute purge time. Helium that represented the carrier gas was calibrated at a flow rate of 1.6 mL/min. The program for column temperature used was set at 8°C/min to 180°C for 10 minutes and subsequently 8°C/min to 240°C for 10 minutes. Inlet temperature was held at 250°C, and the detector temperature was maintained at 260°C, whereas the oven temperature was set to 115°C. Individual compounds in the sample were identified using mass spectral data, by comparing retention time with that obtained for authentic laboratory standards. The peak areas generated were quantified, expressed, and recorded as percentage of total FA detected.

## 3. Results

### 3.1. Qualitative Profile of Bioactives in H. leucospilota and Extraction Efficiency of Solvents

The qualitative profile of bioactive components extracted from sea cucumber* H. leucospilota *organ samples is presented in Table 1. From the table, the presence of saponins, steroids, terpenoids, glycosides, cardiac glycosides, tannins, phlobatannins, phenols, flavonoids, and carbohydrates in different solvent extracts of the sampled sea cucumber organs was confirmed, positive, although in different concentrations.

Among the groups of compounds tested, assessment for phenols, terpenes, and carbohydrates (polysaccharides) was positive in all solvent extracts of all three organs evaluated, using both conventional and Soxhlet extraction procedure (and well distributed in the organic and aqua solvents). On the other hand, test for phlobatannins was negative in all solvent extracts of Cuvierian tubules (CT) and visceral organs (VO) and all other solvent extracts used on body wall (BW), except in acetone (A) and methanol/acetone (MA), indicating that the presence of phlobatannins was restricted to the BW. Screening for tannins was positive in all solvent extracts of both VO and BW but was negative in extracts of CT. Tests for steroids, saponins, cardiac glycosides, and glycosides yielded positive results in solvent extracts of all tissues evaluated except those extracted in A. In the case of flavonoids, screening yielded positive results in all solvent extracts of BW and MA extract of VO. Test for flavonoids, however, yielded negative result in A extract of VO and both solvent extracts of CT.

Results for the quality screening also indicated that the MA solvent combination was by far the most efficient extraction solvent, yielding all the ten groups of compounds across almost all organ samples studied. Conversely, A was observed as the lowest efficient extraction solvent across the organ samples studied, as it was only efficient in extracting 3 groups of compounds in CT (phenols, terpenes, and carbohydrates), 4 groups from VO (phenols, terpenes, carbohydrates, and tannins), and 6 groups from BW (phenols, terpenes, carbohydrates, tannins, phlobatannins, and flavonoids). Comparison between the conventional and Soxhlet extraction procedure from the quality assessment test was in favor of the conventional one (marked in the Table 1 by #), as all the extraction solvents tested on the BW successfully yielded all groups of compounds, except phlobatannins, likely due to the combined use of distilled water that favored the extraction of water soluble bioactives. Consequently, MA and methanol/distilled water (MD) seemed the most efficient solvent combinations for extraction of almost all the bioactives.

### 3.2. Concentrations of Major Groups of Bioactives in H. leucospilota

**Figure 1:**
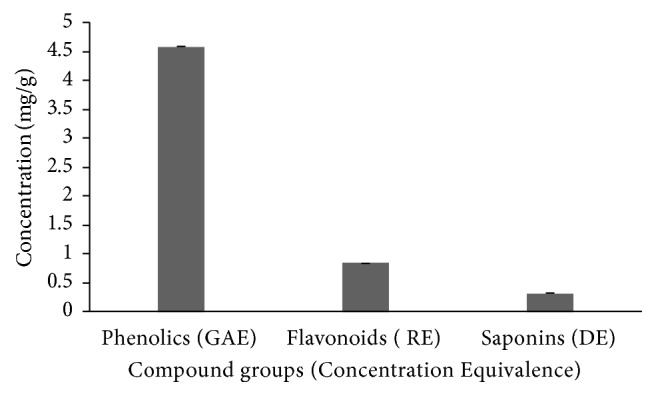
Graphical presentation of estimated concentrations of groups of bioactive components in body wall (BW) extracts of sea cucumber,* H. leucospilota* sampled from Peninsular Malaysia; GAE: gallic acid equivalent, RE: rutin equivalent, DE: diosgenin equivalent (all in mg/g) from study [19].

Table 2 and Figure 1 represent results for total phenolics, saponins, and flavonoids content in BW extracts of sea cucumber,* H. leucospilota *(in different equivalents). The approximate content (% total) is also shown (assuming only the 3 groups of compounds constituted the entire bioactives in the BW of the species). From the table, total phenolics content was measured as 4.58 mg/g, expressed as gallic acid equivalent, GAE, calculated from the standard curve equation: y = 0.0013x, r^2^ = 0.9958. This represented approximately 80% of the 3 groups of bioactives measured in the BW extract.

On the other hand, the content of total flavonoids was measured as 0.84 mg/g, expressed as rutin equivalent, RE, calculated from the standard curve equation:* y* = 0.0009x, r^2^ = 0.996, representing approximately 15% of the bioactives in BW extract of the species. Quantification of total saponins content yielded 0.32 mg/g, expressed as diosgenin equivalent, DE, also calculated from the standard curve equation:* y* = 0.004x, r^2^ = 0.9954, in the BW extract of* H. leucospilota, *with the estimation representing <6% of total of the 3 groups of bioactives evaluated.

### 3.3. Identification and Estimation (% of Compounds Detected) of Bioactives in H. leucospilota

The twelve major subgroups of bioactive components detected in* H. leucospilota *via GC-MS analysis are presented in Table 3. From the table, two ketone compounds were detected, from which, 4-hydroxy-4-methyl-2-pentanone was the major compound detected in all CT, VO, and BW solvent fractions evaluated, with the concentration being the highest in the distilled water fraction of BW. The second ketone, 4-methyl-3-Penten-2-one, was also detected but was limited to only solvent extracts of the CT and VO and was poorly detected in BW aqueous fractions. One phenolic compound was detected, 2, 4-bis (1, 1-dimethylethyl)-Phenol- which was found in most solvent fractions of all organs tested. One of the five alcohols detected, 2-Furanmethanol, was found in most fractions of all organs. Another one (glycerin) was more abundant in aqueous fractions of BW and both organic solvent extracts of VO, but not in CT. The highest concentration of alcoholic compound (trans-9-Hexadec-en-1-ol, 9.15%) was detected in the MA extract of CT. The remaining two alcohols ((+)-trans,trans-5-caranol and 10-Methyl-8-tetradecen-1-ol-acetate) were detected in high concentrations in CT acetone fraction.

Seven different hydrocarbons were isolated and measured in the* H. leucospilota *solvent fractions, two prominent among which were well detected in all solvents: 3-Chlorooctane (which was more in acetone extracts) and 2-Chlorooctane (distributed in A, MA, and aqueous extracts). Two others, 3, 4-dimethyl-1-decene and 1-Nonene, were only detectable in solvent fractions of CT and VO and poorly detected in BW. The remaining hydrocarbons, 3-ethyloctane and Cyclopentane-1-ethyl-3-methyl, were only detectable in MA fraction of CT. For FA, a total of nine were detected by the GC-MS analysis. Of these, only two (n-hexadecanoic acid and Cyclohexane butanoic acid) were detected in solvent fractions of CT, whereas all the other seven were found in the solvent extracts of both VO and BW (distributed in both organic and aqueous fractions). The highest detected FA was n-hexadecanoic acid that was extracted in appreciable concentrations in all solvent fractions of CT, VO, and BW. The vast majority of FA detected in abundance were found in BW aqueous fractions.

Only two carboxylic acids (Acetic and Crotonic acids) were detectable in the GC-MS analysis. Whereas the first was only noted in BW fractions (more in aqua fractions), the second was measured in both fractions of CT and VO. On further analysis, four aldehydes were detected in total, which were mostly not found in the MA and A fractions of CT or the isopropanol/acetone (PA), isopropanol/distilled water (PD), and methanol/distilled water (MD) fractions of BW but were more abundant in the A and MA fractions of VO, as well as MA fraction of BW. Furfural, another aldehyde, was only found in A and MA fractions of BW and 3,7-dimethyl-2,6-octadienal was detected (4.64%) as the most abundant, but only in MA fraction of BW. The most abundantly detected sterol compound (Cholest-3-ene, (5*β*)), followed by Cholesta-3,5-diene, was abundant in solvent fractions (except A) of the 3 organs. Four other sterols detected were found in different solvent fractions of different organs, where 14-methyl-ergost-8-en-ol (3*β*, 5*α*) was the sterol detected in the largest quantity (7.74%) in the PD (aqueous) fraction of BW.

FAMEs and some fatty acid ethyl esters were the group of compounds that was extracted in the highest concentration, being found abundant in especially MA fractions of CT, VO, and BW, but poorly detected in A fraction. The FAMEs that were detected in the highest concentration were five: hexadecanoic acid methyl ester, octadecanoic acid methyl ester, 7-hexadecenoic acid methyl ester, 5,8,11,14-eicosatetraenoic acid ethyl ester (arachidonic acid), and 5,8,11,14,17-eicosapentaenoic acid methyl ester (EPA). Conversely, 4,7,10,13,16,17,19-docosahexaenoic acid methyl ester (DHA) was only found in PA fraction of BW. Interestingly, 11-eicosenoic acid methyl ester, arachidonic acid, and DHA were more abundant in aqueous fractions of BW. Three ester compounds were detected in the sea cucumber extracts, found in CT and VO fractions and only one found in PD fraction of BW (Decanedioic acid, bis (2-ethylhexyl)ester), as the most abundant ester detected was n-tridecyl ester, 2-Propenoic acid (9.1%) in MA fraction of VO. Finally, only 2 amines were extracted and detected (2-methyl-2-Propanamine and 2,4,4-Trimethyl-2-Pentanamine), both of which were not detected in CT but in both fractions of VO and only one in A fraction of BW.

**Figure 2:**
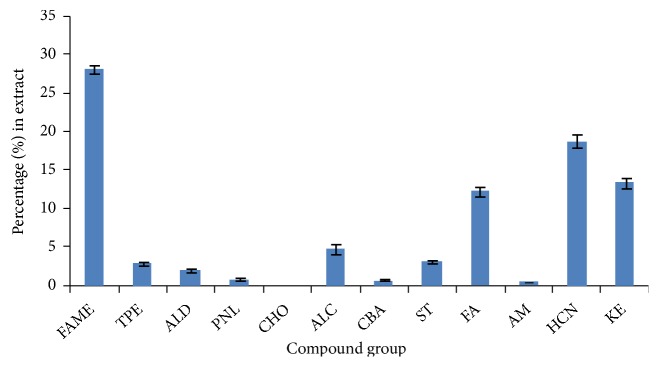
Concentrations (%) of compound subgroups detected via GC-MS and measured in BW extract of sea cucumber,* H. leucospilota*: FAME: fatty acid methyl esters, TPE: terpenes, ALD: aldehydes, PNL: phenols, CHO: carbohydrates, ALC: alcohols, CBA: carboxylic acids, ST: sterols, FA: fatty acids, AM: amines, HCN: hydrocarbons, KE: ketones.

A summarized comparison of the quantitative detection levels of the twelve different subgroups of bioactive compounds in the BW solvent extracts of sea cucumber is shown graphically in Figure 2. From the figure, it would be observed that FAMEs constituted significantly (P<0.05) the most detected compounds and accounted for approximately 30% of all bioactive subgroups. This was followed by hydrocarbons that accounted for over 15% and then ketones and FA that accounted for <15% each. Alcohols, sterols, terpenes, aldehydes, and phenols were all detected within the range of 5% or less. However, the lowest subgroup of compounds detected was carboxylic acids (CBA), followed by amines (AM) and then carbohydrates (CHO), in the decreasing order CBA > AM > CHO, and all 3 were detected within the range of 1% or less of the total bioactives measured.

Overall, when fatty acids and FAMEs (together with fatty acid ethyl esters) are considered as one group of bioactives, this automatically makes them account for approximately 50% of all bioactives in the BW extracts of the sea cucumber species sampled.

### 3.4. Concentration (% of Extracts) of FAMEs in BW Samples of Sea Cucumber via GC-FID Analysis

The GC-FID analysis of the FA detected in three BW solvent extracts of sea cucumber* H. leucospilota* yielded the results presented in Table 4. From the table, of the three different solvent fractions tested (based on relative abundance of PUFA in these solvents in the GC-MS data), saturated FAMEs accounted for 37.07, 57.89, and 53.18% of the FAMEs extracted in ethanol/distilled water (ED), methanol/distilled water (MD), and isopropanol/distilled water (PD) fractions, respectively. Methyl laurate (C12:0), methyl myristate (14:0), methyl palmitate (C16:0), and methyl stearate (18:0) were the principal saturated FA, with C16:0 being the most abundant. Monounsaturated FA, on the other hand, accounted for 45.20, 26.51, and 24.00% of total FAMEs extracted in ED, MD, and PD fractions, respectively, and the principal monounsaturated FA across the 3 solvent extracts was cis-9-oleic methyl ester (C18:1). Polyunsaturated (PUFA) FAMEs were also detected by the solvent extracts used and measured as 15.19, 15.94, and 11.67% in the solvent fractions studied, respectively. The most abundant PUFA FAME was C18:2 (methyl linoleate).

Generally, a comparison among the solvents used on BW extracts revealed that MD and PD were more efficient in extracting saturated FA (57.89 and 53.18%), and ED was more efficient in extracting monounsaturated FA (45.20%), whereas all three solvents exhibited almost equivalent efficiency for extracting PUFA FAMEs from BW of the sea cucumber (15.19, 15.94, and 11.67%, respectively).

## 4. Discussion

### 4.1. Qualitative Analysis for Groups of Compounds

In general, detection of the different bioactives in the different organ extracts evaluated was influenced by the polarity of solvents and sensitivity of the extraction methods employed. It is already well established that highly polar solvents like methanol, ethanol, isopropanol (in lipids), and water (in aqueous medium) have higher capacity to extract high concentrations of polar compounds (such as phenolics, flavonoids, and some saponins) whereas solvents of middle to lower polarity yield different concentrations of bioactives of middle to lower polarity from both plant [31] and animal matrixes [32, 33]. This observation correlates with phenolics, flavonoids, and saponins being the most abundant bioactives (in that decreasing order) extracted in the methanolic, ethanolic, and aqueous fractions of* H. leucospilota *organ extracts. Many other authors have reported different concentrations of these bioactives in different organs and tissues of diverse solvent extracts of sea cucumbers, including phenols and tannins [14, 34], phenols and flavonoids [26]. So far, most marine polyphenols referenced in the literature were isolated and identified as having originated from macroalgae resources [35]; over 8000 different phenolic compounds are currently known [36], and many are likely to be discovered.

Chemical extraction and screening constitute a very useful method to determine the presence and concentrations of bioactive constituents in organisms [37]. Analysis of some isolated bioactive compounds in* H. leucospilota* indicates that they may be completely harmful to humans and other marine organisms, as the substances may have been absorbed into the tissues of the sea cucumber from toxic wastes regularly dumped into the sea, especially heavy metals like Cadmium, Arsenic, and Lead [38]. On the other hand, some other detected substances may help to protect the sea cucumber against harmful bacteria and predators abundant in the marine environment [39, 40]. Consistent with this speculation, a study [41] confirmed that certain isolated organic substances from organs of sea cucumbers were active against both Gram-positive (*Bacillus subtilis*,* Staphylococcus aureus*) and Gram-negative bacteria (*Escherichia coli*,* Klebsiella pneumoniae*) [42], and methanolic extracts of sea cucumbers have also been shown to exhibit cytotoxicity and antifungal activities against* Aspergillus niger* [43]. Furthermore, the shallow reef areas where* H. leucospilota *is commonly found are also home to some predators of the species, making it necessary for the organism to devise strategies to protect itself and may, therefore, justify accumulation or synthesis of protective poisons [44].

### 4.2. Concentration of Compound Groups in BW Extract of H. leucospilota

The presence of phenolics, saponins, and flavonoids in* H. leucospilota *extracts is indication of the medicinal importance of the species. These groups of compounds are known to possess healing properties and have also been demonstrated to be active against several pathogens, thus supporting the traditional use of sea cucumbers for the managing several diseases. Saponins are known to exhibit a wide spectrum of biological effects, including hemolytic, cytostatic, antineoplastic, anticancer, and antitumor activities [45–48]. Studies reported by other authors have also demonstrated the antioxidant properties of sea cucumbers that were described to possess high content of phenolics and flavonoids, where total flavonoids from the gonads, muscles, and digestive tract were noted to constitute the highest repository [8]. Tannins, a group of phenolic metabolites that are found in many terrestrial plants [49], are also known to be abundant in marine resources like macroalgae and sea cucumbers because of their high antioxidant capacity [50].

The largest of the compounds of plants phenolics are flavonoids that account for about 50% of the 8000 naturally occurring phenolic compounds [51]. Many studies have been carried out on terrestrial plants-derived flavonoids, but those on the algal-derived flavonoids are scarce. Consequently, it has been shown that there are basic structural (and likely functional) differences between flavonoids from marine algae and those in terrestrial vegetables and fruits [52], as [53] confirmed that macroalgae are rich sources of the catechins and flavonols, the rare types of flavonoids which sea cucumbers are likely to have in abundance because they feed mainly on seaweeds. These compounds likely constitute part of the approximate quantity (% total, dry weight value) of flavonoids recorded in the present study (14.6%), which was similar to that reported elsewhere (12%) [54].

Saponins, commonly identified as holothurins from sea cucumber with structural features quite comparable to those in Ganoderma, Ginseng, and other medicinally popular tonic herbs, have also been isolated by other authors [55]. This water soluble group of substances is usually found among marine species such as sea cucumbers, star fish, and sponges, with triterpene glycosides reported as predominant among these compounds in sea cucumber. The chemical diversity of triterpene holothurins from sea cucumbers makes them currently the attractive group of compounds targeted in new drugs discovery [56]. Mucopolysaccharides and chondroitin have also been detected in sea cucumbers and have proved useful as nutraceuticals to people suffering from arthritis and connective tissue disorders, to ease joint-pains and arthritis [57].

Detection of phlobatannins in solvent fractions of sea cucumber body walls was also not a surprise. Phlobatannins, which are polymers of phloroglucinol, are known structural components of brown algal cell wall that offer protection to the algae against harmful ultraviolet radiation [35]. Phlorotannins, unlike hydrolysable or condensed tannins, are oligomers of phloroglucinol and are restricted to brown marine algae [58]. They also taste bitter and help the algae to avoid foragers [59]. Sea cucumbers consume macroalgae as their major source of nourishments [13] and may have accumulated the phlobatannins, likely to serve as protection against microbial infection or to also deter predators.

Regardless of their potential, bioavailability is the major challenge confronting researchers when questions about the relationships existing between polyphenols and their health benefits are raised [60]. In other words, the exact* in vivo* effects of consumed polyphenols (either by marine organisms or humans) are yet to be fully understood, as research on the topic is still ongoing, with irregular and vague conclusions between studies when compared to what is known* in vitro*. However, the potential bioavailability of polyphenolics such as phlorotannins from marine algae indicated that over 70% of the consumed plant polyphenols evaluated* in vivo* may be bioavailable and utilizable for their presumed metabolic roles [61].

### 4.3. Bioactive Compounds and Their Concentrations (% of Extract Detected) in H. leucospilota

Data in the literature suggests that the compound 2-pentanone, 4-hydroxy-4-methyl-, one of the compounds found in sea cucumber, has also been isolated from species of red and brown algae (Rhodophyceae and Phaeophyceae) and also in algal species of* Laurencia pinnatifida* and* Pterocladia capillacea*. These substances were reported to be responsible for the antimicrobial and antioxidant activities of these algae [62]. The presence of high concentrations of these compounds in* H. leucospilota* is likely due to dependence of sea cucumbers on phytoplankton and micro- and macroalgae as their major sources of nourishment [13]. Furthermore, the detection of hexadecanoic acid and 5,8,11,4-eicosatetraenoic acid ethyl ester (arachidonic acid ethyl ester; 20:4n6) as other compounds found in high concentration in all extracts was not surprising, as arachidonic acid, a long-chain fatty acid, together with eicosapentaenoic acid (EPA; 20:5n3) and docosahexaenoic acid (DHA; 22:6n3) has been shown to be synthesized by some marine bacteria and phytoplankton and transferred through the food web to other organisms including sea cucumbers that feed on them [63, 64].

These omega 3 and omega 6 FA are essential in human nutrition and are currently highly sought after, even in animal husbandry. The detection of these major components in the tissues of sea cucumber was therefore consistent with its feeding habits as reported by [13], additional reason for its consumption as food. Other bioactive and antiagent substances in sea cucumbers, such as triterpene glycosides, some enzymes, amyloses, fatty acids, and cytotoxins that have potential to improve immunity, resist tumor, offer protection to nerve tissue, reduce pain, and contribute to immune potentiation, anticancer, and anticoagulation activities, have also been properly documented [65].

Revelation by GC-FID analysis that* H. leucospilota *organ extracts contained FAMEs as the most abundant (approximately 50%) subgroup of bioactives, consisting of saturated FA (methyl palmitate, methyl laurate, methyl stearate, and erucic acid methyl ester), monounsaturated FA (*ci*s-9-oleic methyl ester and methyl linoleate), and PUFA (Arachidonic acid, EPA) as some of the important compounds, was expected. The sea cucumber* H. leucospilota *is a bottom feeder on sea sediments that is expected to contain high concentrations of FA. This is because sediments at sea bottoms that the species feeds on are known to contain relatively high contents of branched chain FA [66–68]. The origin of the sediments is believed to be mostly from bacteria, although high levels of algal-derived FA have also been reported in several species of sea cucumbers. Micro- and macroalgae are believed to be the main sources of plant materials in the diet for demersal animals in shallow (<100 m) coastal waters that are grazed directly by herbivorous and omnivorous species [69]. Similarly, micro- and macroalgae are well known as producers of hydrocarbons; consequently, hydrocarbons are expected as constituents of demersal animals that depend on these algae as their sources of energy. On the other hand, ketones found in* H. leucospilota *were also found in certain algal species and may have been transferred through the food web.

Organic matter synthesized in the marine ecosystem by photosynthetic organisms is utilized either directly in the food web or through the microbial loop [70]. Photosynthetic organisms are themselves consumed in the food web and eventually supply essential FA to marine organisms, which are incapable of synthesizing them in sufficient quantities to cover their physiological needs [71]. More pertinently, only plants have demonstrated capacity for* de novo* synthesis of 18:3n-3 and 18:2n-6 FA in marine ecosystems. Consequently, micro- and macroalgae primarily produce omega-3 and omega-6 polyunsaturated fatty acids (PUFA), which are eventually transferred via the food chain to higher animals that feed upon them [72, 73].

Reference [74] opined that the percentage of content of saturated and unsaturated fatty acid esters in animals could also be used as indication of the feeding habits of organisms. The dominance of saturated FA would indicate autotrophic feeding whereas abundance of unsaturated FA indicated heterotrophic feeding, implying that the organism relied more on zooplanktons and small fishes. The later definition is consistent with the FA profile detected in* H. leucospilota. *Monounsaturated fatty acids (MUFA) such as oleic acid and polyunsaturated fatty acids (PUFA) such as arachidonic acid (AA), eicosapentaenoic acid (EPA), and docosahexaenoic acid (DHA) are essential FA required for good human health and nutrition. These FA are required for protection from the risk of coronary heart diseases. They play important roles in the lives of cardiac cells as they are essential fuels for the mechanical and electrical activities of the heart [75]. SFA such as hexadecanoic acid, octadecanoic acid, and tetradecanoic acid were also found in the samples evaluated, although in low concentrations. These FA are known to raise LDL cholesterol and thus increase the risk of coronary heart disease. However, octadecanoic acid (stearic acid) has been shown to have a neutral effect on total blood and LDL cholesterol levels [76–80]. It is not coincidence therefore that the heavy consumption of sea cucumbers over the centuries was for nothing other than the health and nutritional benefits realized by ancient generations, who passed down the habit to their descendants.

Other substances found in the solvent extracts have been demonstrated to have biological activity. For instance, triterpene (a terpenoid) compound was proven to exhibit cytotoxicity against some cancer cells, including human tumor cell lines, as a potential anticancer agent. Another compound, intercedenside A, also demonstrated antineoplastic function against mouse sarcoma and mouse lung cancer [81]. A study in Japan stumbled upon some novel compounds (gangliosides) in a sea cucumber species* H. leucospilota,* which were able to stimulate laboratory-scale nerve cell growth in rat cells [82]. The potential to use compounds like chondroitin sulfate from sea cucumbers for the inhibition of human immunodeficiency virus (HIV) infection has also been proposed, as sulfated polysaccharides, such as fucoidan isolated from seaweeds and sea cucumbers, were reported to exhibit antibacterial, anticancer, and antiviral activities [83]. Polypeptides and polypeptides in sea cucumber extracts have also been shown to exhibit free radical scavenging ability and antioxidant abilities, according to studies reported by [84] and [85], respectively. The number of potential biological activities of compounds extractable from sea cucumbers is still evolving.

### 4.4. FAMEs (% of Extracts Detected) in BW Samples of Sea Cucumber Quantified

Reference [86] concluded that fatty acids of sea cucumber lipids fractions were the key components liable for tissue repair and wound healing properties of sea cucumber. Palmitic acid has been reported to constitute between 20 and 30% of most animal fats and also an important constituent of most vegetable fats (35–45% of palm oil). Stearic acid (octadecanoic acid) is nature's most common long-chain FA found in combined form in natural animal and vegetable fats [87]. In contrast to vegetable oils, however, FA with odd number of carbon (C13:0, 17:0, C19:0) are also found in sea cucumbers, as was also evident from the results of the present study [38]. Among the unsaturated fatty acids* cis*-9-oleic methyl ester (C18:1n9), a MUFA, and methyl linoleate (C18:2n6), an omega-6 PUFA, also formed the major constituents of the lipids.* Cis*-9-oleic methyl ester (*cis*-9-octadecenoic acid) detected as the highest concentration in one of the extracts is reported to be the most abundant MUFA in animal tissues. Methyl linolenate (C18:3n3), an omega-3 PUFA, is commonly found in low concentrations in many animal tissues [15].

Deposit feeding holothurians (among which are sea cucumbers) are reported to possess greater amounts of algal-derived FA such as arachidonic acid, DHA, and EPA. The principal FA of holothurians comprised C20:4 (n-6), C20:1, C20:5 (n-3), C16:0, and C18:0 and also high content of other FA such as C18:1, C20:0, C23:1, and C22:6 (n-3). The results of the present study further showed that more saturated fatty acids were found in* H. leucospilota* compared to the unsaturated FA. Among the PUFA in sea cucumbers, arachidonic acid (C20:4 n-6) is detected to be the principal component in almost all species with relatively higher amounts reported for tropical species, as was observed in the present study [88]. This FA is known to play an important role in the processes of cell growth and clotting of blood, leading to healing of wounds [79] and lending support to the long-time utilization of sea cucumbers in Asia as traditional remedy to treat burns and cuts [89].

High level of arachidonic acid (AA) was detected in* H. leucospilota *via GC-MS analysis. AA is known to be responsible for blood clotting [90] and was the main PUFA detected in all the samples evaluated. In tropical holothurians the level of AA usually prevails appreciably over that for EPA [16]. Oleic acid, the major MUFA on the other hand, is associated with the prevention of coronary heart disease and was found to be the highest in GC-FID analysis. The major omega-3 FA found in* H. leucospilota* point to its importance and use in traditional medication for cure and in the prevention of many illnesses and infections. The results, therefore, indicate that* H. leucospilota* is particularly rich in MUFA, PUFA, and amino acids that compete with levels found in some of the commercially important species of sea cucumber, in terms of nutritional and medicinal values.

## 5. Conclusion

In conclusion, from the results of the investigations carried out in this study, it is clear that* H. leucospilota* is a potential source of many bioactive, nutritionally and physiologically important compounds that could be utilized in nutraceutical and pharmaceutical industries as sources of both food and medicine. However, further studies need to be carried out to isolate, purify, and specifically investigate the efficacies and biological activities of all the identified compounds, and the current focus on the species to mine for new drugs is justified.

## Figures and Tables

**Table 1:** Qualitative biochemical analysis of crude extracts of different organ samples of *H. leucospilota *in various solvents.

Compound Groups	Organs in Solvent Extracts									
	A/CT	MA/CT	A/VO	MA/VO	A/BW^#^	MA/BW^#^	PA/BW^#^	ED/BW^#^	MD/BW^#^	PD/BW^#^
Phenols	+^#^	+^#^	+^#^	+^#^	+^#^	+^#^	+^#^	+^#^	+^#^	+^#^
Terpenes	+^#^	+^#^	+^#^	+^#^	+^#^	+^#^	+^#^	+^#^	+^#^	+^#^
Carbohydrates	+^#^	+^#^	+^#^	+^#^	+^#^	+^#^	+^#^	+^#^	+^#^	+^#^
Phlobatannins	-	-	-	-	+^#^	+^#^	-	-	-	-
Tannins	-	-	+^#^	+^#^	+^#^	+^#^	+^#^	+^#^	+^#^	+^#^
Steroids	-	^#^	-	+^#^	-	+^#^	+^#^	+^#^	+^#^	+^#^
Saponins	-	+^#^	-	+^#^	-	+^#^	+^#^	+^#^	+^#^	+^#^
Cardiac glycosides	-	+^#^	-	+^#^	-	+^#^	+^#^	+^#^	+^#^	+^#^
Glycosides	-	+^#^	-	+^#^	-	+^#^	+^#^	+^#^	+^#^	+^#^
Flavonoids	-	-	-	+^#^	+^#^	+^#^	+^#^	+^#^	+^#^	+^#^

^*∗*(+)^Present,( ^−^)Absent; A/CT (acetone/Cuvierian tubule), MA/CT (methanol-acetone/Cuvierian tubule), A/VO (acetone/visceral organ), MA/VO (methanol-acetone/visceral organ), A/BW (acetone/body wall), MA/BW (methanol-acetone/body wall), PA/BW (isopropanol-acetone/body wall), ED/BW (ethanol-distilled water/body wall), MD/BW (methanol-distilled water/body wall), and PD/BW (isopropanol-distilled water/body wall)[19].

**Table 2:** Concentrations of groups of bioactive components in body wall (BW) extracts of sea cucumber, *H. leucospilota* sampled from Peninsular Malaysia from a study by [19].

Compound group	Concentration of Sample	Approximate
	(mg/g)	Quantity
		(% total)
Phenolics	4.58 ± 0.002 mg GAE/g	79.79
Flavonoids	0.84 ± 0.000 mg RE/g	14.63
Saponins	0.32 ± 0.002 mg DE/g	5.58

^a-^Values represent mean of 3 replicate determinations ± SD.

GAE: gallic acid equivalent (mg GAE/g); RE: rutin equivalent (mg RE/g); DE: diosgenin equivalent (mg DE/g).

**Table 3:** Bioactive components detected and measured via GC-MS indifferent solvent extracts of body organs of sea cucumber, *H. leucospilota *sampled from Peninsular Malaysia.

RT	Compounds	% of Compounds in extract									
		A/CT	MA/CT	A/VO	MAVO	A/BW	MABW	PABW	EDBW	MDBW	PDBW

	*Ketones*										

5.558	4-hydroxy-4-methyl-2-Pentanone,	37	15	18	8.69	27	18.9	2.5	17.7	30.8	34.1
5.533	4-methyl-3-Penten-2-one	0.5	3.82	1.8	1.17	1.9	-	-	-	-	1.22

	*Phenols*										

20.225	2,4-bis(1,1-dimethylethyl)-Phenol-	2.7	1.11	2	1.33	5.9	2.14	-	0.95	1.41	1.73

	*Alcohols*										

13.367	2-Furanmethanol	3.7	1.05	4.7	0.53	1.5	0.42	-	0.18	0.67	0.72
20.783	(+)-trans,trans-5-caranol	3.8	-	-	-	-	-	-	-	-	-
21.017	10-Methyl-8-tetradecen-1-ol-acetate	6.6	-	-	-	-	-	-	-	-	-
22.6	trans-9-Hexadec-en-1-ol	-	9.15	-	-	-	-	-	-	-	0.18
20.35	Glycerin	-	-	0.73	0.5	-	-	0.97	0.49	6.54	-

	*Hydrocarbons*										

5.642	3-Chlorooctane	5.8	3.37	20	4.13	20	4.75	-	1.2	-	3.67
5.808	2-Chlorooctane	6.8	10.5	9.5	2.26	11	4.5	-	3.66	7.28	8.36
8.933	3,4-dimethyl-1-decene	2.5	4.02	3.9	-	1	1.14	-	-	-	-
9.075	3-ethyloctane	-	5.45	-	-	-	-	-	-	-	-
5.592	1- methyl -3-ethyl Cyclopentane	-	11.7	-	-	-	-	-	-	-	-
5.592	Cyclopentane, 1-ethyl-2-methyl	-	-	-	-	5.19	-	-	-	-	-
7.692	1-Nonene	1.1	1.17	-	0.59	0.8	-	-	-	-	-

	*Fatty acids*										

23.117	Tetradecanoic acid	-	-	2	0.21	1.2	-	1.16	1.04	0.93	0.65
25.783	n-Hexadecanoic acid	0.8	0.52	8.8	1.12	6	1.39	7.93	12.7	5.68	3.43
26.217	Z-11-Hexadecanoic acid	-	-	5.7	-	4.1	-	-	-	-	-
26.267	9-Hexadecenoic acid	-	-	3.5	-	2.7	0.79	4.8	3.24	2.41	1.67
29.683	*cis*-9-Octadecenoic acid (oleic acid)	-	-	0.45	0.77	0.5	-	2.94	1.52	-	-
24.533	Eicosanoic acid (arachidic acid)	-	-	0.17	0.51	-	-	1.32	0.68	0.22	-
27.133	Heptadecanoic acid	-	-	-	-	0.5	-	-	1.72	-	-

RT	Compound	% of Compounds in extract									
		A/CT	MA/CT	A/VO	MA/VO	A/BW	MA/BW	PA/BW	PD/BW	ED/BW	MD/BW

	*Fatty acids continued.*										

10.642	Cyclohexane butanoic acid	-	1.06	-	-	-	-	-	-	-	-
28.9	Octadecanoic acid	-	-	1.6	0.2	1.9	0.57	3.54	3.72	1.33	0.85

	*Carboxylic acid*										

10.65	Acetic acid	0.8	-	-	-	0.8	0.36	-	0.82	1.1	0.97
14.717	Crotonic acid (2-butenoic acid)	0.4	0.22	0.52	0.67	0.46	-	-	-	0.16	0.4

	*Aldehydes*										

15.267	2,4- dimethyl benzaldehyde	-	-	1.3	0.83	2.6	0.85	-	0.44	0.91	-
14.225	3,7-dimethyl-2,6-Octadienal	-	-	-	-	-	4.64	-	-	-	-
10.35	Furfural	-	-	-	-	1.8	0.83	-	-	-	-
22.033	(Z)-9- Octadecenal	-	-	-	1.11	1.3	-	-	-	-	-

	*Sterols*										

40.9	Stigmast-5-en-3-ol, (3*β*, 24*σ*)- (sitosterol)	-	-	0.5	-	2.54	-	-	-	2.15	
47.017	14-Methyl-ergost-8-en-ol, (3*β*,5*α*)-	-	-	-	-	-	-	0.77	7.34	-	-
38.558	Stigmasta-5,22-dien-3-ol, acetate, (3*β*, 22*ζ*)-	-	-	-	0.85	-	-	-	1.72		
31.9	Ergosta-14,22-dien-3-ol, acetate, (3*β*, 5*α*, 22*ε*)-	-	-	-	-	-	-	-	-	1.03	-
30.9	Cholest -3-ene, (5*β*)	-	1.08	-	-	3.6	3.16	-	-	3.37	3.56
33.858	Cholesta-3,5-diene	-	0.95	-	0.43	0.8	2.63	-	-	2.29	1.96
37.717	Ergosta-4, 22-diene	-	-	-	-	-	1.98	-	-	-	-
35.592	Ergosta-4,6,22-triene	-	-	-	-	0.4	-	-	-	-	1.21

	*Fatty acid methyl/ethyl esters*										

19.592	9-Hexadecenoic acid methyl ester	-	3.36	0.32	12.8	1.3	3.61	-	-	-	-
19.308	Hexadecanoic acid methyl ester	-	7.86	-	17.63	-	9.05	0.24	-	-	-
21.225	Octadecanoic acid methyl ester	0.6	7.41	0.44	8.35	1.1	4.82	-	-	0.25	0.33
21.483	13- Octadecenoic acid, methyl ester	-	1.96	-	3.8	-	1.72	-	-	-	-
23.15	7-Hexadecenoic acid methyl ester	-	3.91	-	2.12	-	2.12	-	0.13	-	0.17
17.783	Pentadecanoic acid methyl ester	-	0.33	-	4.07	-	1.72	-	-	-	-
19.575	Hexadecenoic acid methyl ester	-	-	-	-	-	6.35	-	-	-	-

RT	Compound	% of Compounds in extract									
		A/CT	MA/CT	A/VO	MA/VO	A/BW	MA/BW	PA/BW	PD/BW	ED/BW	MD/BW

	*Fatty acid methyl/ethyl esters continued*										

22.292	Eicosanoic acid methyl ester	-	1.7	-	-	-	1.07	-	-	-	-
34.875	11-Eicosenoic acid methyl ester	-	0.44	0.2	0.75	-	0.14	21.7	12.1	6.07	3.59
17.233	Methyl tetradecanoate	0.9	0.87	-	3.37	-	1.21	-	-	-	-
39.717	5,8,11,14-Eicosatetraenoic acid ethyl ester (arachidonic acid)	-	8.27	-	10.5	1.1	12.7	20.6	11.9	6.04	3.02
40.725	5,8,11,14,17- Eicosapentaenoic acid, methyl ester (EPA)	-	2.55	-	5.57	-	3.68	-	-	-	-
42.842	4,7,10,13,16,17,19-Docosahexaenoic acid, methyl ester (DHA)	-	-	-	-	-	-	2.74	-	-	-

	*Esters*										

31.783	Decanedioic acid, bis (2-ethylhexyl) ester	4	-	-	2.8	-	-	-	-	-	1.14
24.883	Hexanedioic acid, bis (2-ethylhexyl) ester	-	-	3.8	-	-	-	-	-	-	-
17.075	n-tridecyl ester, 2-Propenoic acid	-	9.1	-	-	-	-	-	-	-	-

	*Amines*										

16.017	2-methyl-2-Propanamine	-	-	3.1	0.85	-	-	-	-	-	-
16.308	2,4,4-Trimethyl-2-Pentanamine	-	-	-	-	2.8	-	-	-	-	-

- represents not detectable; A/CT: acetone Cuvierian tubule; MA/CT: methanol/acetone Cuvierian tubule; A/VO: acetone visceral organ; MAVO: methanol/acetone visceral organ; A/BW: acetone body wall; MA/BW: methanol/acetone body tissue; PABW: isopropanol/acetone body tissue; EDBW: ethanol/distilled water body wall; MDBW: methanol/distilled water body wall; PDBW: isopropanol/distilled water body wall extracts.

**Table 4:** Quantitative profile of major FAMEs detected via GC-FID and measured in body wall (BW) solvent extracts of sea cucumber, *H. leucospilota *sampled from Malaysian peninsular.

Fatty acid name	No. of C-atoms	Tissue extract		
(% of total extract)		EDBW	MDBW	PDBW
methyl octanoate	C8:0	1.64 ± 0.00	0.00 ± 0.00	0.00 ± 0.00
methyl decanoate	C10:0	0.00 ± 0.00	0.43 ± 0.22	0.00 ± 0.00
methyl laurate	C12:0	10.41± 1.67	0.604 ± 0.09	0.00 ± 0.00
methyl tridecanoate	C13:0	0.00 ± 0.00	0.00 ± 0.00	0.00 ± 0.00
methyl myristate	C14:0	4.04 ± 0.00	0.886 ± 0.00	10.71 ± 0.47
methyl pentadecanoate	C15:0	0.64 ± 0.00	0.048± 0.001	0.808± 0.02
methyl palmitate	C16:0	14.81 ± 0.66	44.86 ± 3.39	26.477± 0.62
methyl heptadecanoate	C17:0	0.379 ± 1.73	0.138 ± 0.01	0.885 ± 0.02
methyl stearate	C18:0	4.83 ± 0.41	9.516 ± 1.82	6.835 ± 0.22
methyl arachidate	C20:0	0.321 ± 0.00	1.314 ± 0.14	1.189 ± 0.08
methyl behenate	C22:0	0.00 ± 0.00	0.09 ± 0.04	0.33 ± 0.16
erucic acid methyl ester	C23:0	0.00 ± 0.00	0.00 ± 0.00	5.949 ± 0.30
*Total Saturate d FA* (%*total FA)*		*37.07*	*57.89*	*53.18*
cis-9-oleic methyl ester	C18:1	45.0 ± 7.08	26.38 ± 3.28	22.29 ± 0.47
trans-9-elaidic methyl ester	C19:1	0.00 ± 0.00	0.00 ± 0.00	0.00 ± 0.00
methyl eicosenoate	C20:1	0.20 ± 0.00	0.134 ± 0.01	1.71 ± 0.26
*Total mono un saturate d FA* (%* total FA)*		*45.20*	*26.51*	*24.00*
methyl linoleate	C18:2	14.79 ± 3.51	15.57 ± 1.63	11.107 ± 0.14
methyl linolenate	C18:3	0.40 ± 0.00	0.368 ± 0.02	0.566 ± 0.07
*Total PUFA (*%*total FA)*		*15.19*	*15.94*	*11.67*

^a^Values represent mean of 3 replicate determinations ± standard deviation.

EDBW: ethanol/distilled water; MDBW: methanol/distilled water; PDBW: isopropanol/distilled water.

## Data Availability

The data and materials supporting the conclusions of this article are included within the article.
